# Bibliometric analysis of human monkeypox research from 1975 to 2022 and novel prevention and control strategies

**DOI:** 10.3389/fpubh.2022.995965

**Published:** 2022-09-27

**Authors:** Jiyong Lin, Guiyu Li, Peiling Zhong, Qiang Zeng, Lei Liu, Liang Chen

**Affiliations:** ^1^Department of Infectious Diseases, Shenzhen Traditional Chinese Medicine Hospital, Shenzhen, China; ^2^Department of Traditional Chinese Medicine, The Eighth Affiliated Hospital, Sun Yat-sen University, Shenzhen, China

**Keywords:** monkeypox, VOSviewer, bibliometric analysis, novel prevention and control strategies, infectious disease

## Abstract

**Background:**

Since human monkeypox was reported, many related literatures have been published. This study aimed to evaluate the research hotspots and future development trends of human monkeypox by a bibliometric analysis, to analyze the preventive and control measures of various countries in response to human monkeypox outbreaks.

**Methods:**

The Web of Science Core Collection database was searched for all monkeypox related literature published from 1975 to 2022, and the search strategy was “TS = monkeypox.” Bibliometric analysis was performed using VOSviewer software based on retrieval data. Contribution metric methods and visualization were used to analyze the top issues in the field of human monkeypox.

**Results:**

From 1975 to 2022, a total of 1,068 monkeypox research papers were included, of which American researchers published 663 papers, and it was also the country that participated in the most international cooperation. Centers for Disease Control Prevention USA is the most prolific institution and a leader in research collaborations. The Journal of Virology has the largest number of published papers on monkeypox. In addition, Damon Inger K has made significant contributions to monkeypox research, with both the most published and the most citation. A total of 2,847 keywords were identified, four top topics were obtained through cluster analysis: (1) human monkeypox epidemiology and species research. (2) human monkeypox virus vaccine and experimental research. (3) human monkeypox disease diagnosis and treatment studies. (4) human monkeypox disease prevention and immunization studies. To curb the spread, regions or countries have developed and implemented detailed managements. The prevention and control measures focus on the isolation of suspected or confirmed patients, the investigation and tracking of the source of the disease, the disposal of pollutants, vaccination and the protection of health workers.

**Conclusions:**

The number of human monkeypox literature has grown since 2003. Infection, vaccine and efficacy were the top topic over the past 47 years while the contact tracing, testing, surveillance and vaccination have been the major concerns since the human monkeypox outbreak in May 2022. The treatment and management of human monkeypox deserves further attention.

## Introduction

Human monkeypox is a viral zoonotic disease caused by the monkeypox virus, which is capable of transmission between animals and humans, and secondary transmission between humans. Its animal hosts include a range of rodents such as squirrels, Gambian kangaroos, dormouse and non-human primates. The clinical manifestations are included fever, superficial lymph node enlargement, skin and mucous membrane rash, and in severe cases, multiple organ failure and death may occur (1, 2). The human monkeypox outbreak in 2022 was first detected in the UK on May 7, 2022. World Health Organization (WHO) issued a disease information bulletin and assessed the global public health risk for human monkeypox as moderate on May 29, 2022 (3).

Bibliometric analysis is a new scientific method for evaluating the contributions of a field of research, including those of countries, institutions, authors, and journals. It can predict top topics and trends in a certain research field through information visualization (4, 5). However, there are few bibliometric studies in the field of monkeypox research.

In this study, we conducted a bibliometric analysis of monkeypox literature published in the core journal of the Web of Science Core Collection database, including the number of annual publications, countries contribution, international collaborations, institutions, journals, authors. In order to clarify the trends and hotspots of monkeypox research, keyword co-occurrence overlay visualization graph analysis was carried out. We analyzed the prevention and control measures of various countries or regions in response to human monkeypox outbreaks. We hope this study can provide a new perspective and reference for future human monkeypox research and prevention.

## Materials and methods

The global literatures about monkeypox published between 1975 and 2022 were scanned in the Web of Science collection database. The search strategy was: “TS = monkeypox.” The information for the documents that meet the requirements contained year of publication, language, journal, title, author, affiliation, keywords, document type, abstract, and counts of citation which were exported into TXT format. The date of the retrieval was 18th Jun 2022.

We used VOSviewer software (www.vosviewer.com) to analyze all keywords, as well as collaborations between countries, organizations, and authors, related to research on monkeypox. The visual analysis steps of VOSviewer software were as follows, started the VOSviewer software and clicked “Create Map” to enter Choose types of data, selected “Create a map based on bibliographic date,” clicked next to enter Choose data source, selected “Read date from bibliographic database files,” enter Select files, and imported documents in Web of Science files, clicked next to enter choose type of analysis and counting method for visual analysis. All data were analyzed using Microsoft Excel 2003 (Microsoft Corp., Redmond, WA, USA). Bar graphs were using OriginPro 2018 (OriginLab, Northampton, MA, USA). Visualization map was performed using Arcmap 10 (ArcGIS, ESRI, Redlands, CA, USA).

## Results

### Bibliometric analysis of publication output

Totally 1,068 publications on the topic of monkeypox were identified in the Web of Science collection database between 1975 and 2022, which included 766 (71.7%) original research papers, 108 (10.1%) review papers, 57 (5.3%) editorials, and 302 other forms of publications including letters, case reports, etc. Almost all the publications (1,036, 97.0%) were written in English, followed by 14 publications in French.

### Bibliometric analysis of annual publications and the citations

**Figure 1:**
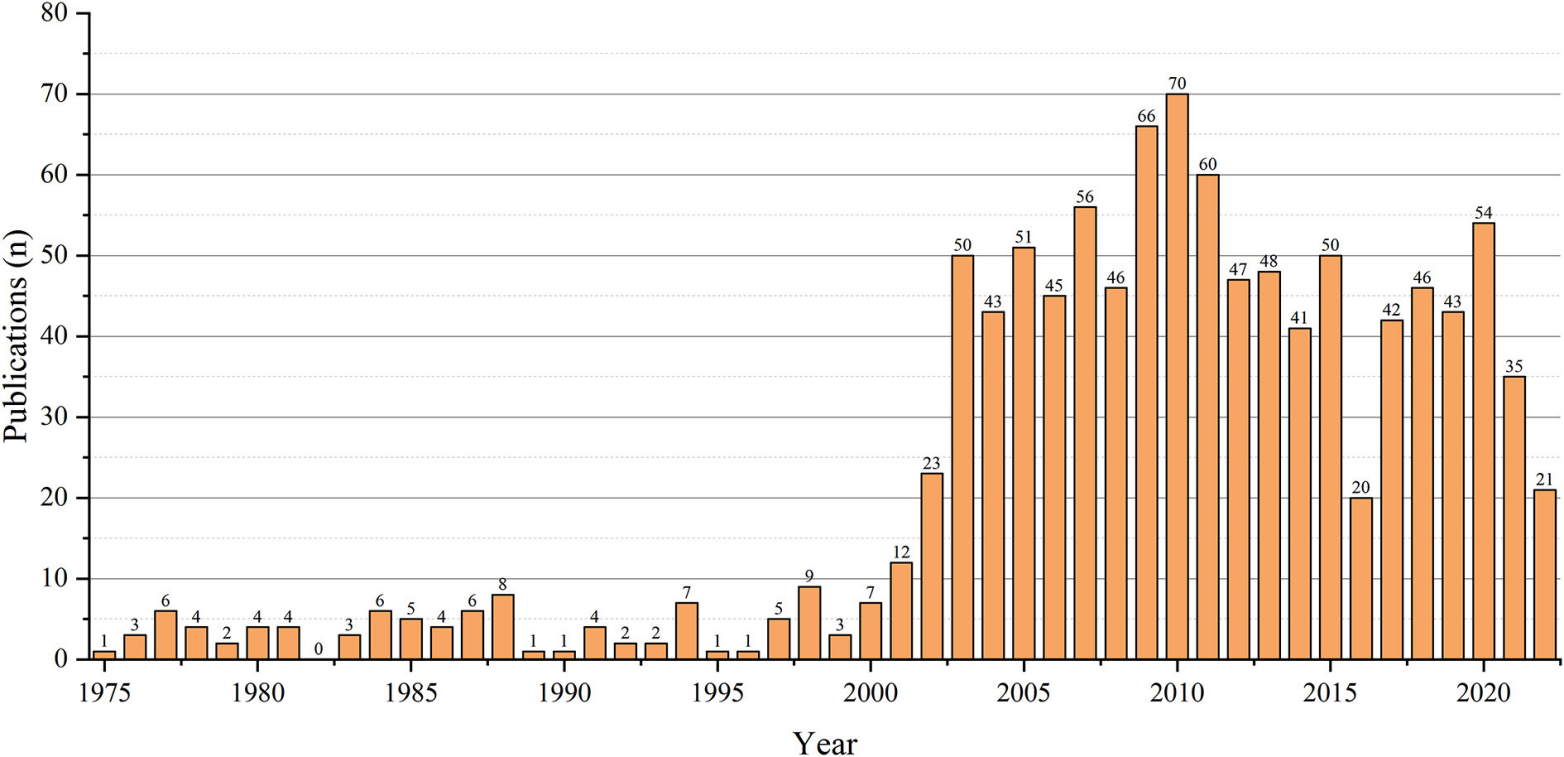
Annual publications on monkeypox research.

From 1975 to 2022, Web of Science indexed 1,068 papers on monkeypox. The results from Web of Science showed that from 1975 to 2001, the number of publications on monkeypox remained stable, however from 2003 to the present saw a significant increase in publications (Figure 1).

The top 20 most cited papers for monkeypox were listed in Supplementary Table S1. Among them, 18 papers were published after 2003. It mainly introduces monkeypox virus clinical research and disease transmission research, including case report and descriptive research, monkeypox treatment, and prevention research.

### Bibliometric analysis of themes and trend topics

**Figure 2:**
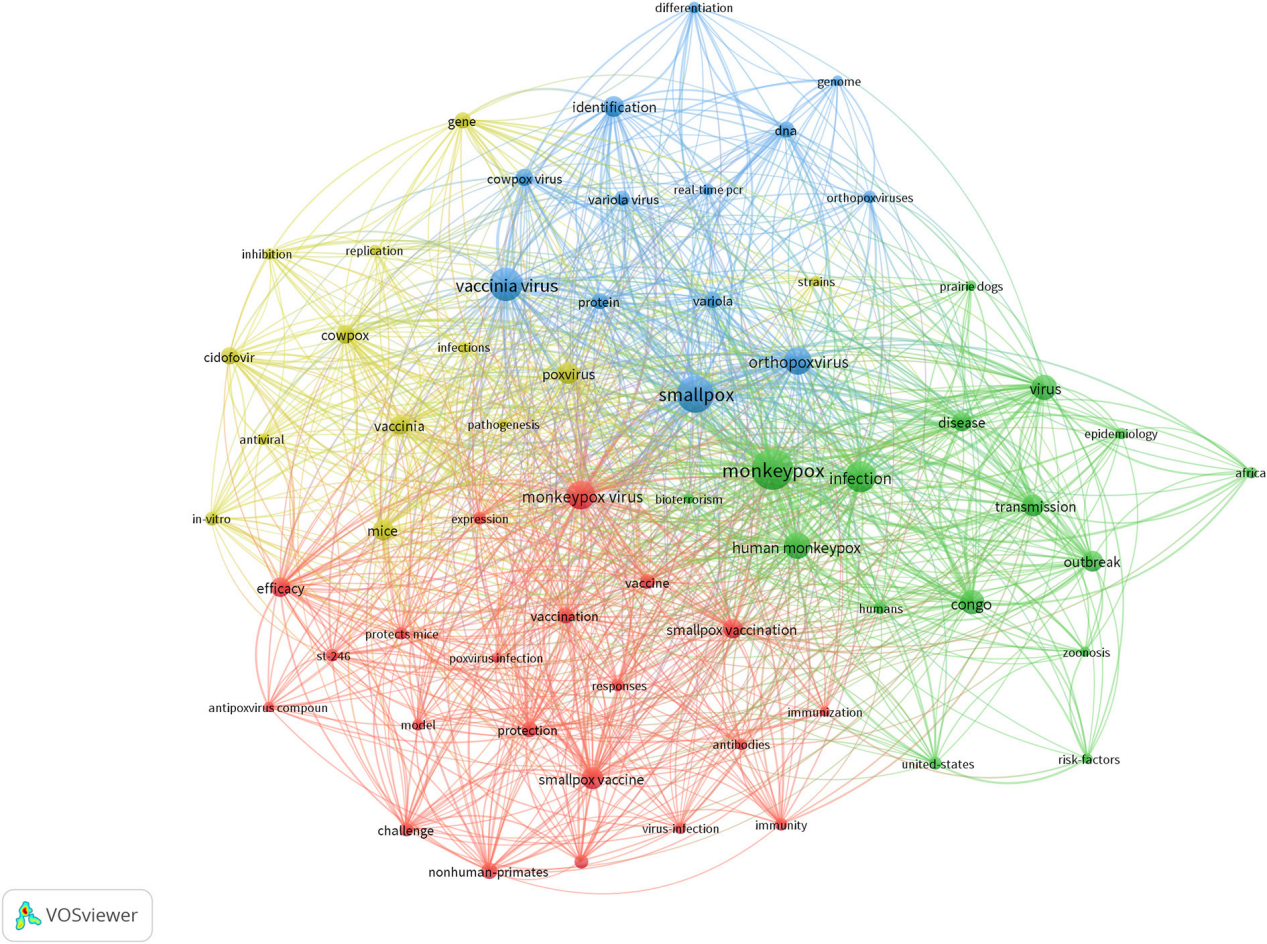
Research hotspots in the field of monkeypox.

A total of 2,847 hotspots were retrieved from Web of Science, and the total frequency of hotspots appeared 9,126 times. Table 1 showed the top 20 top topics. Smallpox (*n* = 213), vaccinia virus (*n* = 175), infection (*n* = 158) were the leading keywords, except monkeypox. As indicated in Figure 2, four themes of monkeypox studies were found. The blue cluster involved monkeypox disease area and species. The green cluster involved monkeypox virus vaccine and experiment research. The red cluster involved monkeypox disease diagnosis and treatment. The yellow cluster involved monkeypox disease prevention and immunization.

**Table 1:** Keywords of monkeypox.

**Number**	**Keywords**	**Frequency**	**Percent (%)**
1	Monkeypox	268	2.94
2	Smallpox	213	2.33
3	Vaccinia virus	175	1.92
4	Infection	158	1.73
5	Monkeypox virus	136	1.49
6	Orthopox virus	122	1.34
7	Human monkeypox	113	1.24
8	Virus	103	1.13
9	Congo	97	1.06
10	Poxvirus	81	0.89
11	Transmission	80	0.88
12	Smallpox vaccine	79	0.87
13	Mice	73	0.80
14	Outbreak	73	0.80
15	Identification	72	0.79
16	Efficacy	70	0.77
17	Smallpox vaccination	70	0.77
18	Vaccinia	70	0.77
19	Disease	66	0.72
20	Cowpox	62	0.68

### Bibliometric analysis of authors

**Figure 3:**
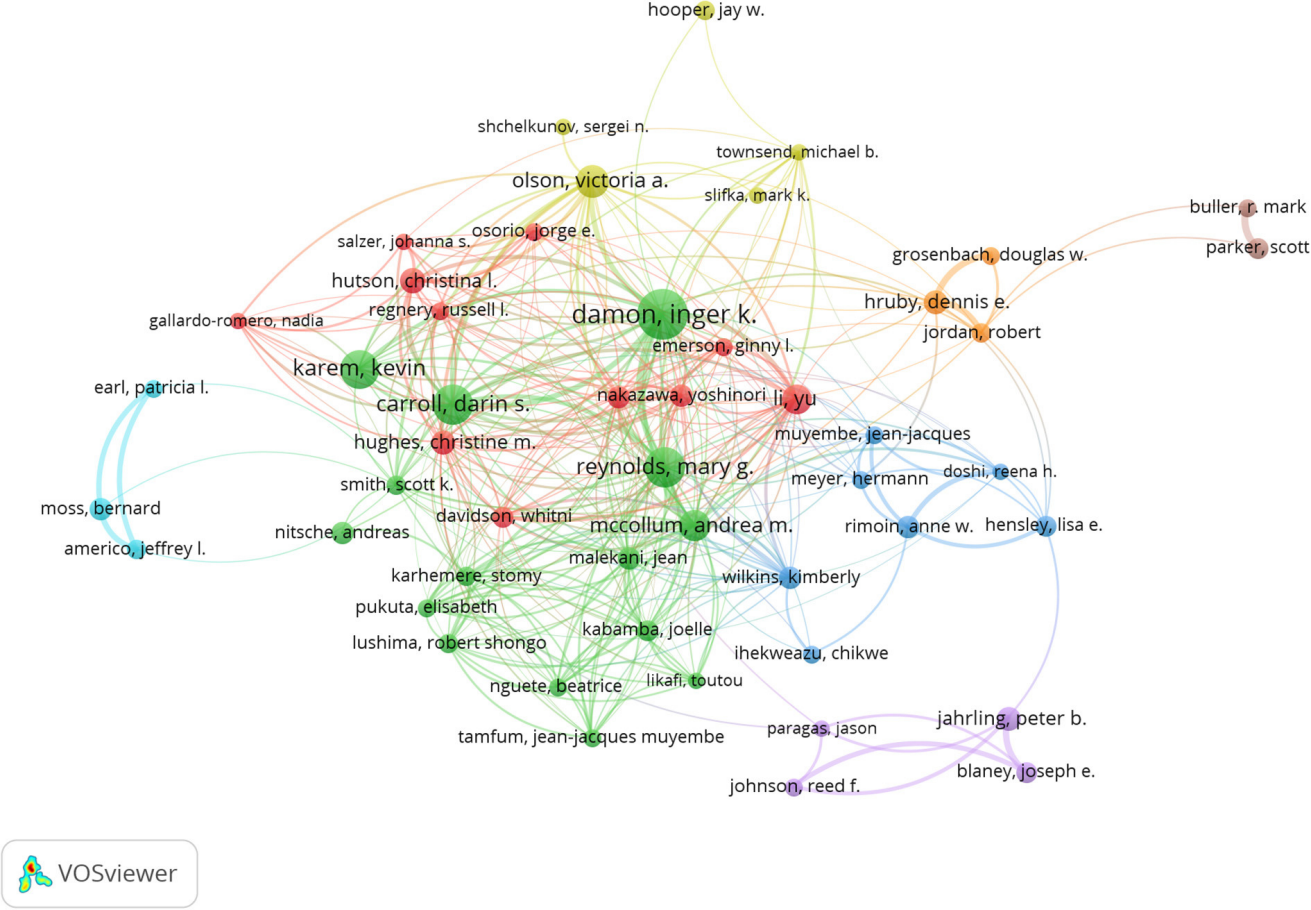
Author co-authorship overlay visualization map in the field of monkeypox. The author's minimum document number threshold was set to 10, and 50 authors with high-frequency publications were screened.

**Figure 4:**
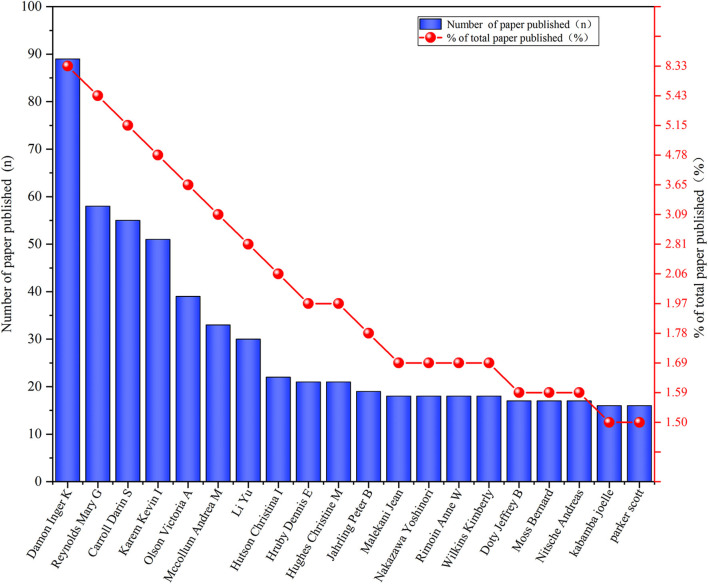
Top 20 authors with most published papers in the field of monkeypox. The Y-axis on the left indicated the number of publication and the right indicated the proportion of the total number.

**Figure 5:**
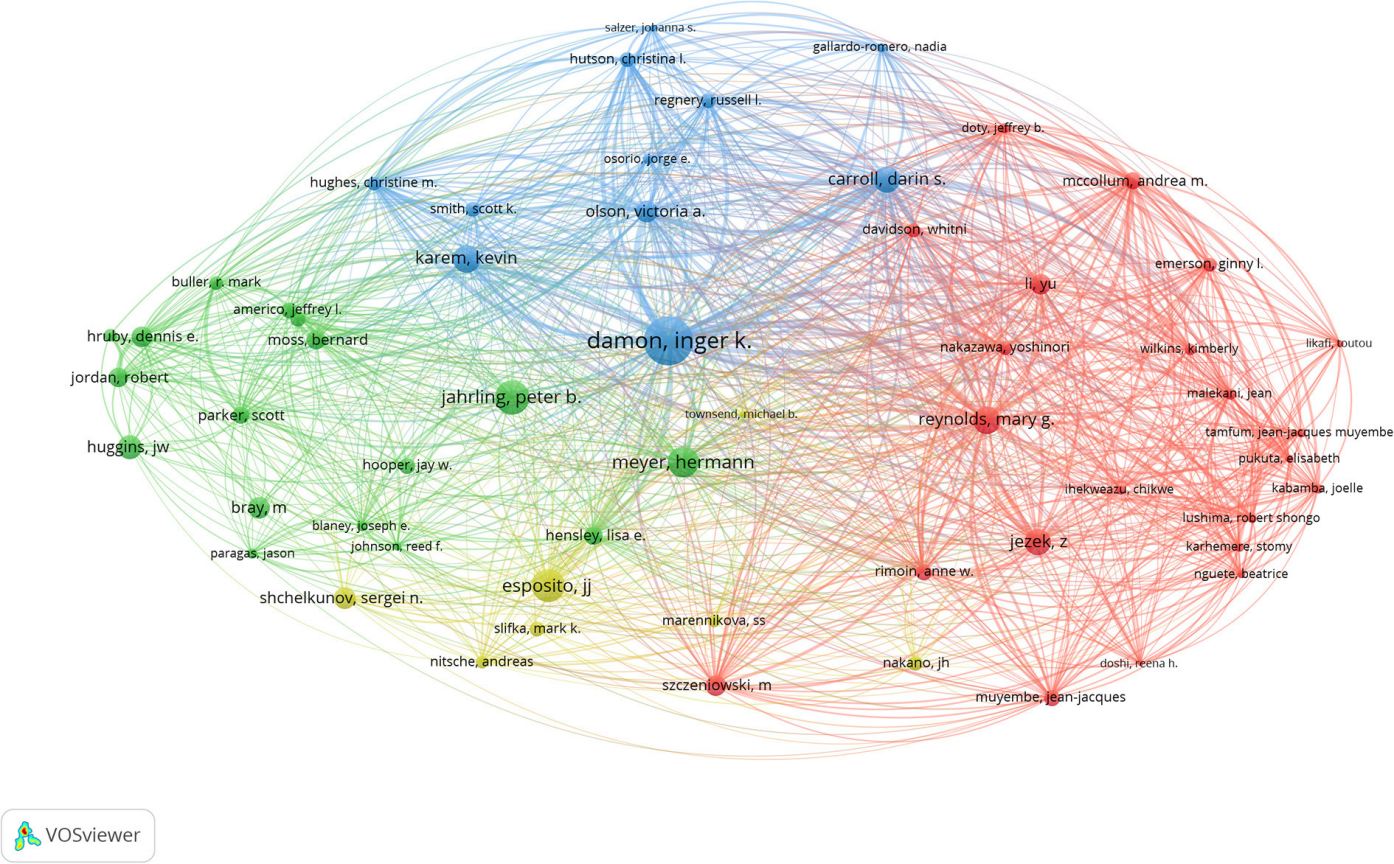
Author co-citation overlay visualization map in the field of monkeypox.

**Figure 6:**
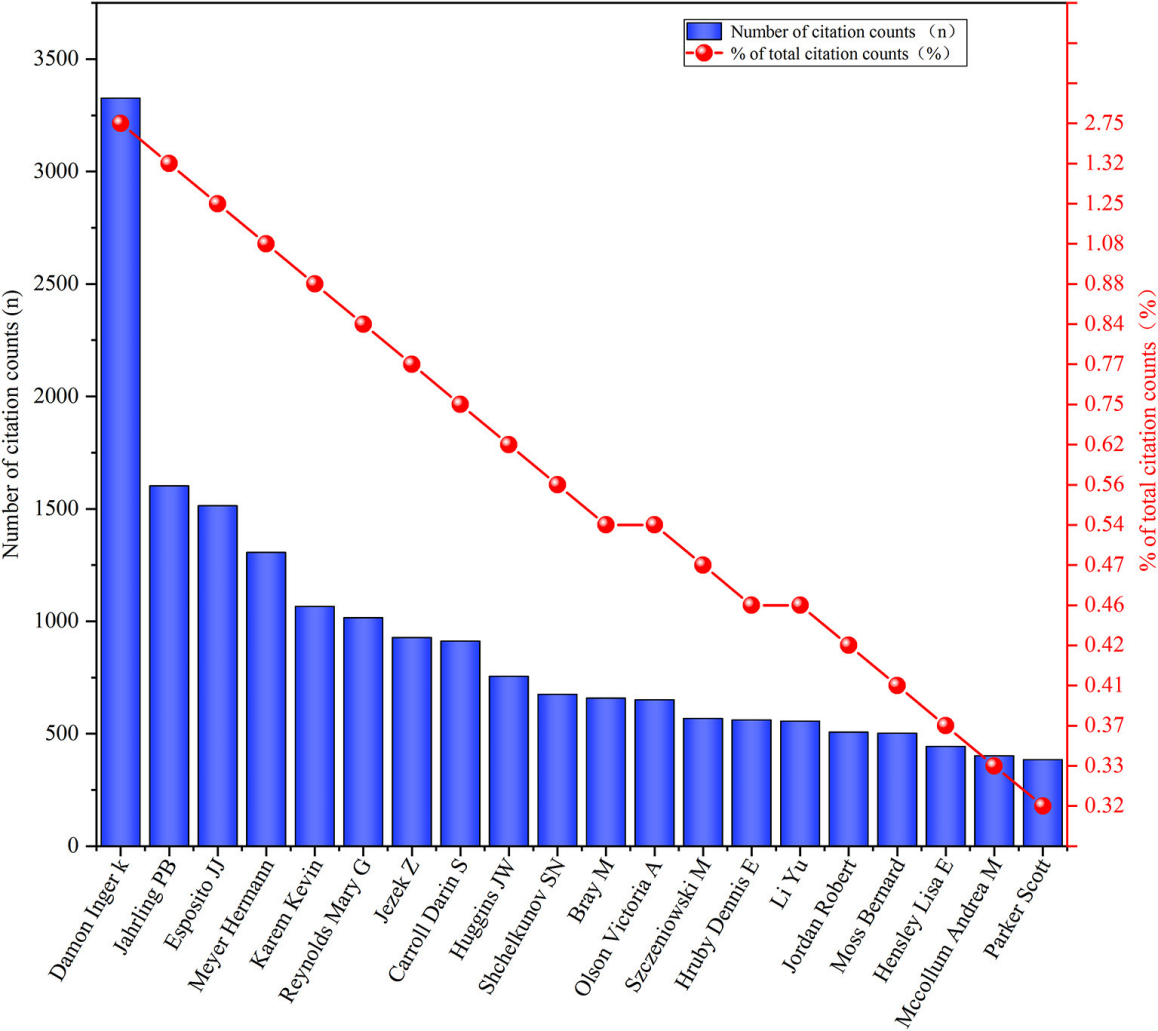
Top 20 authors with most-cited papers in the field of monkeypox. The Y-axis on the left indicated the number of citation and the right indicated the proportion of the total number.

The visual map of author overlays was using the VOSviewer software (Figure 3). The top fifth authors with the most published papers were Damon Inger K (*n* = 89), Reynolds Mary G (*n* = 58), Carroll Darin S (*n* = 55), Karem Kevin I (*n* = 51), and Olson Victoria A (*n* = 39). The top 20 authors by the number of published documents and the proportion of the total number were shown in Figure 4. The author's citation analysis was also generated, 57 authors were identified, and the top fifth cited authors were Damon Inger K (*n* = 3,327), Jahrling PB (*n* = 1,602), Esposito JJ (*n* = 1,514), Meyer Hermann (*n* = 1,306), Reynolds Mary G (*n* = 1,016) (Figure 5). The top 20 cited authors and the proportion of the total number were as shown in Figure 6.

### Bibliometric analysis of countries/regions and institutions

**Figure 7:**
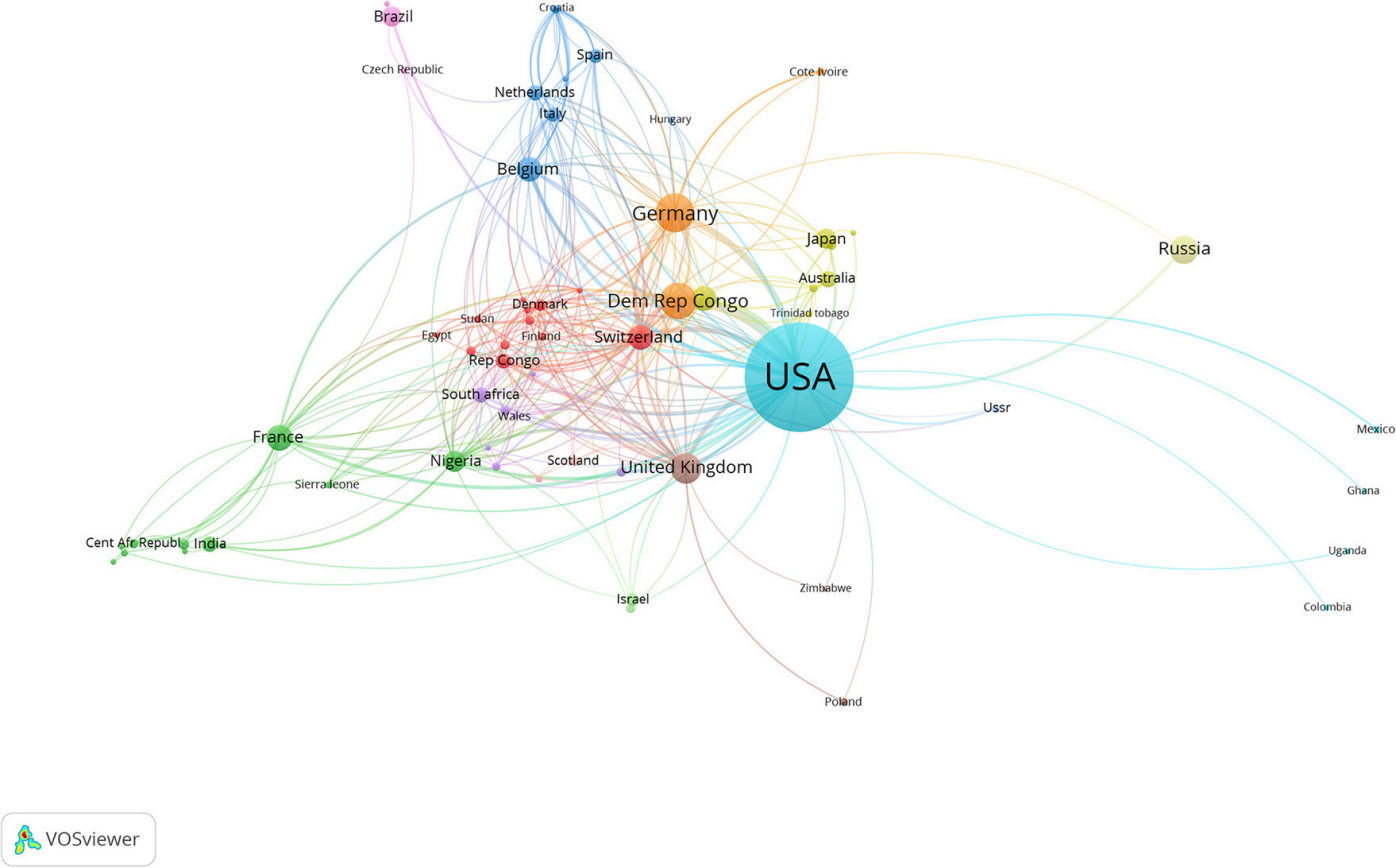
The distribution of countries or regions in monkeypox research.

**Figure 8:**
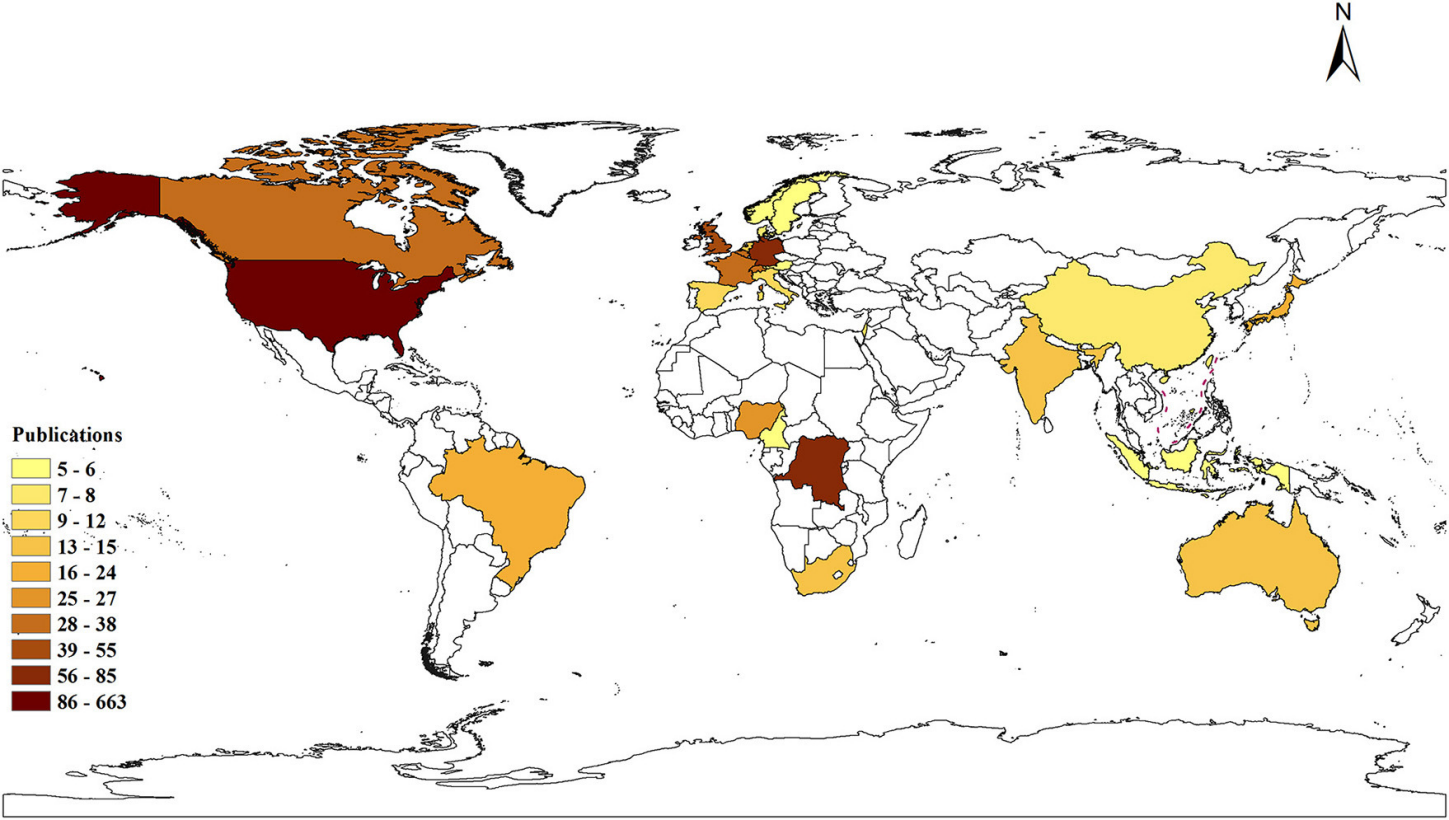
Global distribution of monkeypox literatures. The color of each country or region on the world map represents the number of literature published, according to the color gradient in the lower left corner.

According to the Web of Science Core Collection database, 76 countries or regions contributed to publications on monkeypox. The top 22 countries or regions in terms of the number of publication (*n* ≧ 5) on monkeypox were presented on a world map in Figures 7, 8, and the top 20 were presented as numbers in Table 2. Close cooperation between countries or regions was very common in the world, and the United States was the country that participates most frequently in international cooperation.

**Table 2:** Top 20 countries or regions contributing to publications in monkeypox research.

**Number**	**Country/region**	**Publications**	**Percent (%)**
1	USA	663	47.73
2	Germany	85	6.12
3	Dem Rep Congo	78	5.62
4	UK	55	3.96
5	Russia	46	3.31
6	France	38	2.74
7	Canada	36	2.59
8	Belgium	35	2.52
9	Switzerland	34	2.45
10	Nigeria	27	1.94
11	Brazil	24	1.73
12	Japan	23	1.66
13	Australia	15	1.08
14	Netherlands	14	1.01
15	South Africa	14	1.01
16	Rep Congo	13	0.94
17	India	13	0.94
18	Spain	12	0.86
19	Italy	12	0.86
20	Israel	10	0.72

**Figure 9:**
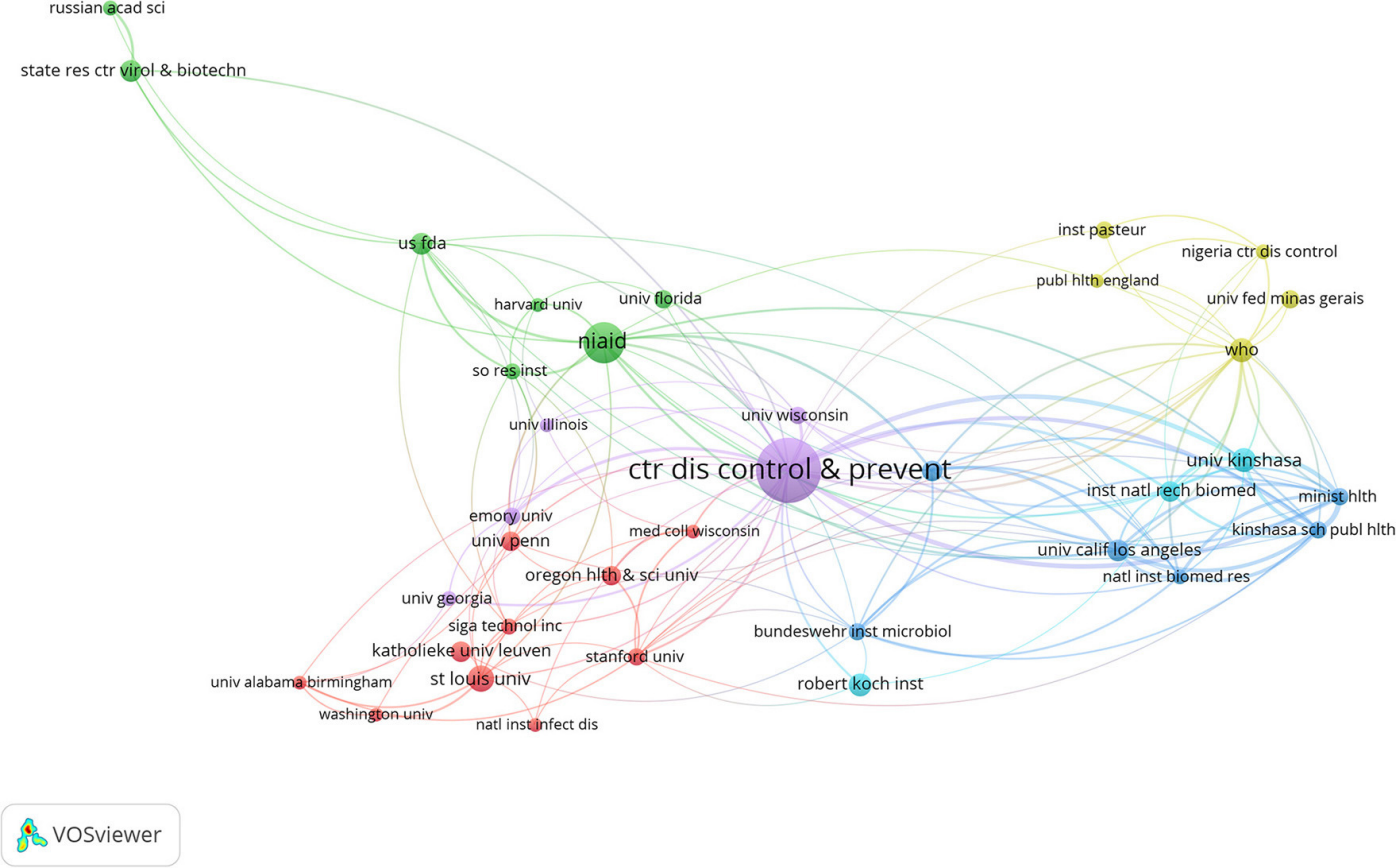
Co-authorship overlay visualization map of institutions.

We then assessed the most productive institutions. As shown in Supplementary Table S2 with 165 papers published, the Centers for Disease Control Prevention USA was the most productive institution, followed by NIH National Institute of Allergy and Infectious Diseases (*n* = 71), Saint Louis University (*n* = 37), Universite de Kinshasa (*n* = 31), World Health Organization (*n* = 29). Among the top five most productive institutions, the Universite de Kinshasa was in Dem Rep Congo, World Health Organization was in Switzerland and the rest were from the United States. The collaborative network was generated using VOSviewer software, with a threshold of 10 as the minimum number of documents for an institution. Thirty-six out of 1,049 institutions were identified (Figure 9). In this study, the Centers for Disease Control Prevention USA published 165 papers with 4,838 citations (Supplementary Table S2), and collaborated with almost all influential scientific institutions in monkeypox research (Figure 9).

### Bibliometric analysis of the bibliographic coupling

**Figure 10:**
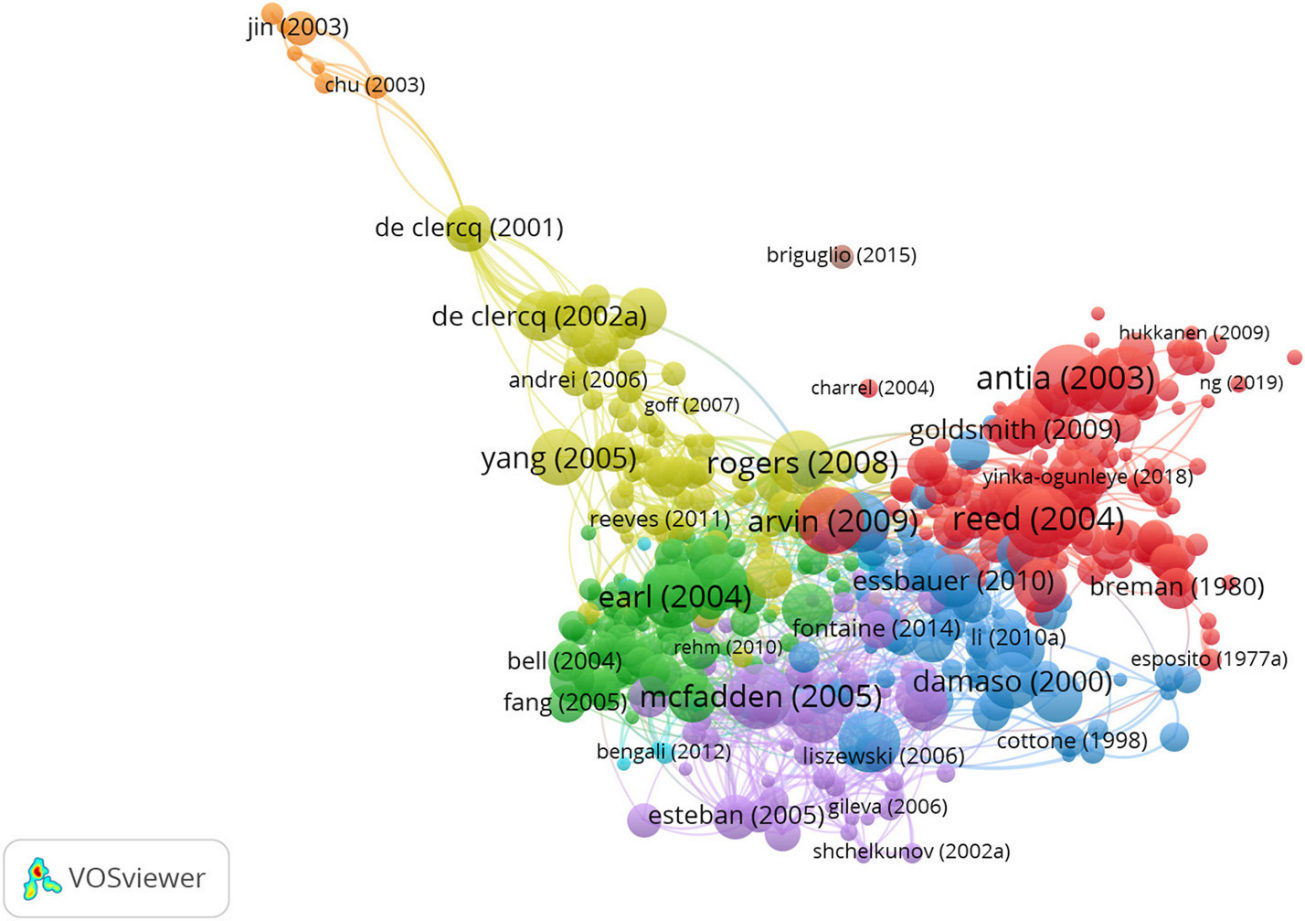
Bibliographic coupling of documents in the monkeypox research. The minimum number of related documents was set to 10, and eight clusters were obtained from the analysis.

The bibliographic coupling diagram of the document was shown in Figure 10. Cluster 1 includes 123 items, and the research field was clinical features (red part). This representative paper was published by Reed in the New England Journal of Medicine in 2004. Cluster 2 has 87 projects mainly related to monkeypox virus epidemiology (green). Cluster 3 has 86 projects mainly related to monkeypox virus vaccine defense (blue). Cluster 4 has 77 projects focusing on monkeypox virus related treatment measures (yellow). Cluster 5 has 72 projects mainly related to clinical detection of monkeypox virus (purple). Cluster 6 has 50 projects mainly for monkeypox virus proteomics research (sky blue). Cluster 7 has 4 projects mainly related to the environmental infection of monkeypox virus dark yellow). Cluster 8 has two items discussing the relationship between antiviral drugs and monkeypox (brown).

**Figure 11:**
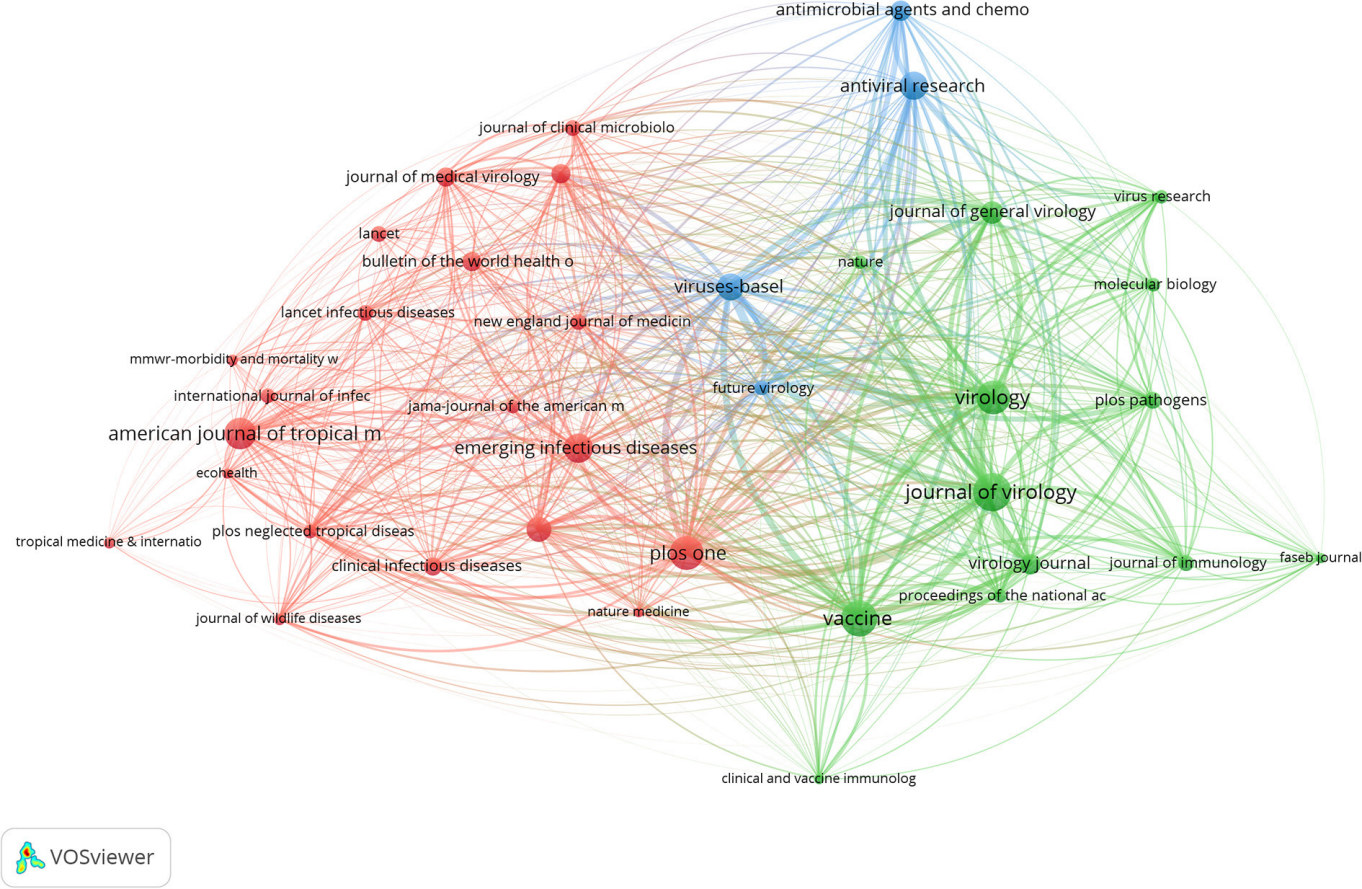
Bibliographic coupling of sources in monkeypox research. Different colors indicated different research areas, the size of the circles indicated the number of co-citations, and the distance relationship between the two circles indicated their relevance.

The bibliographic coupling diagram of the source was shown in Figure 11. The minimum number of related documents was set to five, and three types of clusters were obtained by analysis. The first three groups of sources were Journal of virology, Vaccine, and Virology.

### Analysis of prevention and control strategies

**Figure 12:**
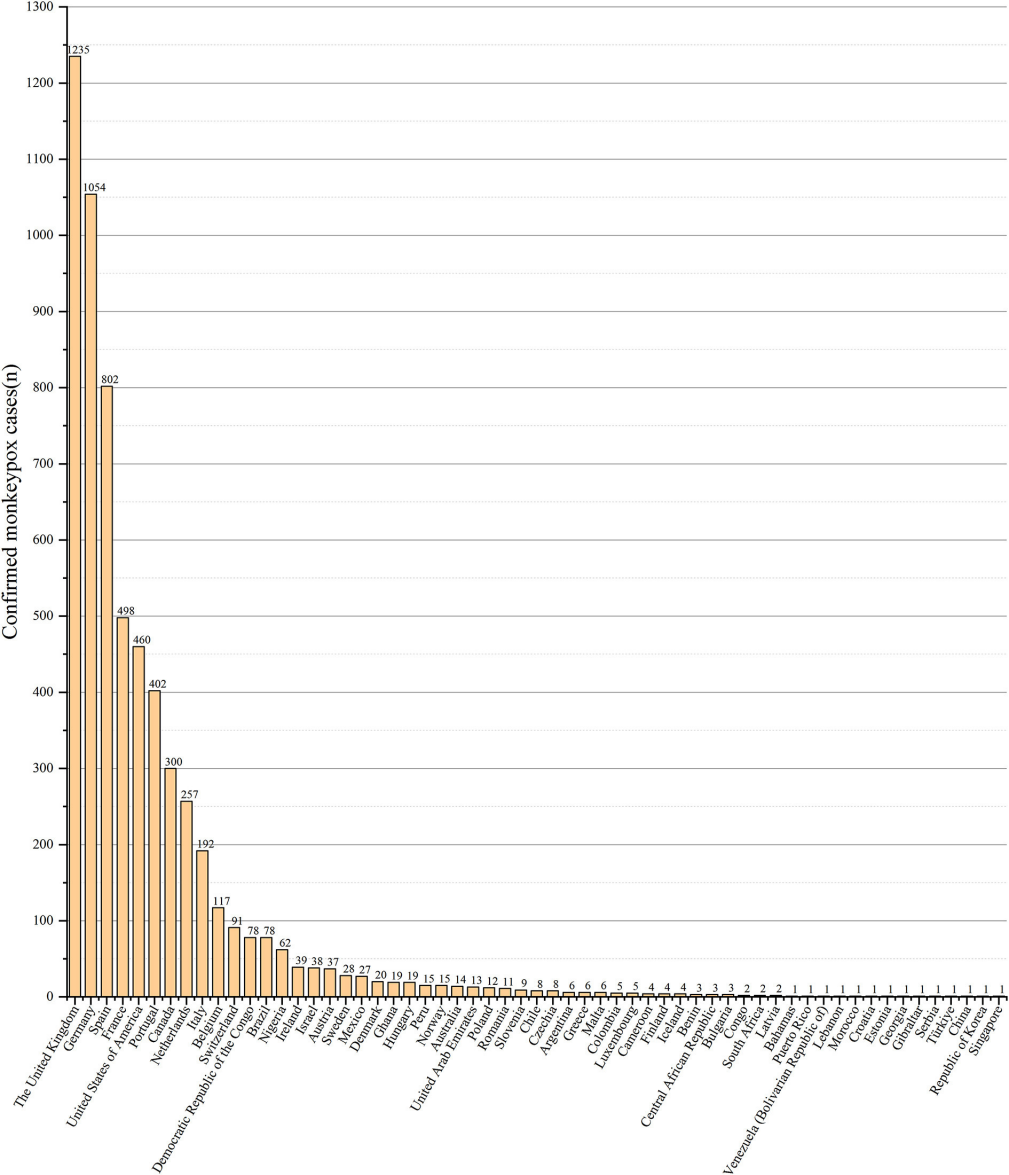
Confirmed human monkeypox cases reported by regions/countries from January 1 to July 1, 2022. The data were derived from World Health Organization (https://www.who.int/).

From early May 2022, 59 countries have reported more than 6,000 cases (6). Human monkeypox cases have increased rapidly since May 2022, especially from the number of reported cases in the UK (Figure 12). Countries have also actively responded to WHO's call to initiate public health emergencies. They reported probable and confirmed cases of human monkeypox to WHO on a weekly basis. Various regions or countries have developed and implemented detailed measures to curb the spread of human monkeypox (Table 3). The prevention and control measures focus on the isolation of suspected or confirmed patients, the investigation and tracking of disease sources, the disposal of pollutants, vaccination, and protection training for health workers. Controlling the source of infection and conducting contact tracing and investigation are commonly used measures to control human monkeypox. Africa is working to strengthen laboratory diagnosis and surveillance of monkeypox. Although vaccination is an effective means of prevention and control, due to the limited supply of local vaccines, vaccination is only recommended for high-risk groups in Africa. In the face of the human monkeypox outbreak in Spain, the prevention and control measures taken include finding the source of infection, close contacts, controlling the route of transmission, and preparing vaccine supply reserves. The Portuguese government quarantined those infected with human monkeypox and close contacts. Unlike other countries and regions, the Portuguese government is not immediately encouraging vaccination, but is evaluating the pros and cons of vaccination. Although the Chinese mainland has not yet found a human monkeypox case in 2022, it has formulated prevention and control measures, including publicizing the transmission route of human monkeypox, preventive measures, quarantine of confirmed cases and close contacts, and import restrictions.

**Table 3:** Public health advice/management by regions/countries.

**Region/country**	**Public health advice/management**
WHO (6)	• Adequate screening space, ventilation, environmental cleaning and disinfection. • Enhanced Rash-like disease surveillance and case search in primary and secondary health care facilities. • Train health workers and other healthcare workers in contact with suspected or confirmed cases to take infection control precautions. • Investigate and isolate suspected cases and provide supportive care, and consider preparing medicines and vaccines under investigation protocols. • Samples collected from suspected cases or suspected animals are to be processed by trained staff in an appropriate laboratory. • Any symptoms of returnees from monkeypox endemic areas should be reported to health workers, including recent travel history and vaccination history. • Avoid contact with dead animals (rodents, marsupials, and primates) that may carry monkeypox virus, and avoid eating and handling game. • Maintain hand hygiene and use personal protective equipment.
Africa (7)	• Enhance surveillance and laboratory diagnostics to detect cases and stop the silent spread of the virus. • Targeted vaccination is recommended for those who have been exposed to monkeypox or are at high risk, including health workers, laboratory personnel and outbreak team responders.
The United Kingdom (8)	• Isolation of confirmed cases. • Contact tracing of confirmed cases. • Assess exposure risk of contacts and self-quarantine high-risk contacts for 21 days and be told to quarantine for 21 days if necessary. • Conduct detailed investigations of contacts to identify sources of infection and chains of transmission. • Vaccinate high-risk contacts to reduce the risk of symptomatic infection and serious illness.
Spain (9)	• Proactively look for infected people and advise contacts to self-isolate at home instead of mandatory quarantine if they have a fever or any related symptoms, and contact relevant medical staff. • Search for people who have been in contact with an infected person. • Avoid contact with wild or domestic animals, pets must be excluded from the patient's environment. • Vaccines have been ordered.
Portugal (10)	• Infected patients are recommended to be quarantined. • People who were not in their place of residence at the time of diagnosis are advised not to travel until the quarantine period ends. • Confirmed cases avoid contact with wild or domestic animals. • Tracing close contacts. • Close contacts will not be quarantined, but must self-monitor their temperature daily for 21 days after exposure and minimize social interaction by constantly wearing masks. • Close contacts with fever or other symptoms consistent with symptoms of illness should immediately self-isolate at home and be monitored. • The pros and cons of vaccination are being studied.
France (11)	• Vaccination should be offered to contacts of adults at risk of confirmed or suspected cases. • Confirmed cases should be investigated to determine the most likely infection. • Contact tracing. • Advise at-risk contacts to monitor their temperature twice daily for 3 weeks following the last at-risk contact with a suspected or confirmed case. • Suspected cases are advised to self-isolate for 21 days.
United States of America (12)	• Investigate reported confirmed cases. • Medical institutions at all levels pay close attention to patients with suspected monkeypox rash and report them in a timely manner. • Facilitating access to vaccines and treatments for those most at risk of contracting or severe illness.
China (13)	• Actively and widely publicize the knowledge of monkeypox virus prevention and control, so that the public can take preventive measures in a timely and effective manner. • Manage sources of infection. • Contact tracing and investigation. • Self-health monitoring of returnees from epidemic areas. • Restrict the import of African rodents and primates to reduce the risk of domestic spread of the virus. • Suspected and confirmed cases should be strictly isolated in single room and strictly disposed of pollutants. • The close contacts of suspected and confirmed cases should be registered, quarantined and medically observed. The medical observation period is 21 days.

## Discussion

The global outbreak of the COVID-19 virus has not completely disappeared, and there have been renewed concerns about the emergence of multiple cases of human monkeypox virus infection (14). Human monkeypox, a rare infectious disease caused by the monkeypox virus, occurs mainly in some African countries, but cases have recently emerged in at least 12 countries outside Africa (15).

In this study, in terms of the number of papers published annually, before 2001, the number of papers published per year was less than 10, mainly in the Congo region of Africa. After 2003, the number of published papers have jumped, with more than 50 papers published per year in most years. The increase in the number of published papers may be related to the outbreak of human monkeypox in many states in the United States in 2003 (16). Among the top 20 cited papers, 18 were from 2003 and later, and most of the papers came from the United States. In our study, Damon Inger K had both the highest number of published monkeypox-related papers and the highest number of total citations (Figures 4, 6). In addition, Jahrling PB, Esposito JJ, Meyer Hermann, Karem Kevin I collaborated closely to produce a large number of highly cited publications, as evidenced by the citation visualization graph (Figure 5). Therefore, those researchers may be a leader in the field of monkeypox research.

Our study showed that monkeypox research mainly focuses on the frequent occurrence of keywords such as smallpox, vaccinia virus, orthopox virus, infection, Congo, disease identification, efficacy, smallpox vaccine, etc., (Figure 2). It suggested that research on monkeypox disease prevention and control strategies will still be a research hotspot in the next few years. Scientists are still trying to fully understand monkeypox, and we hope that in the near future scientists will make breakthroughs in monkeypox defense and treatment.

The United States was the country with the largest number of monkeypox publications, accounting for 47.3% The top three countries with the most publications were Germany and Dem Rep Congo, highlighting their influence in the field of monkeypox research. Centers for Disease Control Prevention USA was considered to be the most prolific institution and the most frequently cited institution, and cooperated with almost all influential scientific research institutions in the field of monkeypox research, including the NIH National Institute of Allergy and Infectious Diseases and Saint Louis University. These results indicated that international cooperation in human monkeypox research was frequent and will still be the trend in the future. Our results showed that the Journal of Virology, Vaccine, and Virology were among the top three journals. The journal of virology has published the most papers related to monkeypox, and it was also the most frequently cited, suggesting that human monkeypox played an important role in virus-related papers.

According to WHO reports of confirmed cases of human monkeypox, countries in the European Region and the Americas Region were currently reporting the highest number of cases. Studies have shown that monkeypox virus has abnormal mutations. Monkeypox virus nucleotides usually have only 1–2 mutations per locus per year, while the human monkeypox virus outbreak in 2022 has nearly 50 genetic mutations, making the virus easier to spread. Among the human monkeypox virus genome samples from the 2022 outbreak, 15 single nucleotide polymorphism sites, secondary variants and gene deletions appeared, and these microevolutions showed signs of better adaptation to the human body (17). Countries such as Europe and the Americas have gradually eased restrictions on entry after prolonged periods severe restrictions due to the COVID-19 epidemic, leading to closer contact between countries around the world, which was one of the factors that has contributed to the spread of the human monkeypox epidemic. Most of the human monkeypox cases reported so far have occurred in men, mostly among people who self-identify as gay, bisexual, and other men who have sex with men on social and sexual networks in urban areas. A higher degree of regional openness is reported in Europe. The basic reproductive number (R0) was higher than 1 in MSM (Men who have Sex with Men) populations and lower than 1 in other populations, as estimated by mathematical models. R0 was estimated to be 1.8 in Spain, 1.6 in the UK, and 1.4 in Portugal (18). At present, there were no confirmed human monkeypox patients in mainland China in 2022, which may be due to the government's strict new crown entry quarantine measures and the active promotion of human monkeypox prevention and control knowledge to the public.

Contact tracing, testing, and surveillance are key to help better determine the extent of the current outbreak and break chains of transmission. In order to reduce infectivity of human monkeypox, 21-day quarantine for suspected or confirmed cases was adopted and recommended. Since the typical incubation period of human monkeypox virus is 5–21 days, 21-day quarantine is a necessary measure to reduce contagion. Human monkeypox is a self-limiting disease, and most patients recover without drug treatment. Currently, symptomatic treatment is recommended for human monkeypox treatment. Certain antiviral drugs have been shown to be effective against human monkeypox infection. The European Medicines Agency (EMA) approved tecovirimat, an antiviral drug for human monkeypox, in 2022, based on data from animal and human studies (19). The drug is not yet widely available, and more data on its efficacy and safety are brewing. Therefore, vaccination remains the primary prevention and control measure. The Spanish government, faced with a local human monkeypox outbreak, first ordered vaccines to prevent and control the outbreak. Vaccination against the now eradicated smallpox virus has been shown to have 85% cross-protection against human monkeypox (20). The first-generation smallpox vaccine used in the smallpox eradication program in the 1970s no longer exists. The development of second- and third-generation smallpox vaccines was driven by concerns about smallpox as a biological weapon. The safety of these new vaccines has improved (21). The U.S. Food and Drug Administration (FDA) has approved two vaccines in the United States, ACAM2000 (IMVAMUNE) and JYNNEOS (IMVANEX), for the prevention of monkeypox infection (22). However, the safety and efficacy of new vaccines against monkeypox still require further research, especially in people with severe immune system problems, and therefore need to be evaluated by policymakers. This may also be the reason why the Portuguese government did not actively encourage vaccination in the first place, but first evaluated the vaccine. At present, although some countries or regions have different measures for vaccine selection and vaccination population. However, due to limited vaccine supply, prioritizing vaccination of high-risk groups is recommended. Especially in Africa, where vaccines are extremely scarce, early detection and early isolation can only be achieved by strengthening monkeypox laboratory diagnosis and monitoring to control the spread of monkeypox. In addition, it is also necessary to actively publicize the knowledge of monkeypox transmission and prevention methods to the public as one of the methods of monkeypox prevention. The Chinese mainland, although no human monkeypox cases have been found during the human monkeypox outbreak in various countries in 2022, as a country with a large population, it has formulated detailed prevention and control measures to deal with possible dangers, including publicizing human monkeypox prevention knowledge to the public, restricting imports, as well as monitoring people returning from human monkeypox outbreaks and quarantining possible and confirmed cases.

There are several limitations in this study. First, the literature data sources for this study are limited to the Web of Science core database, which may not be able to comprehensively assess the development trend of monkeypox. We hope to expand to more databases such as Scopus or PubMed in the near future. Second, due to the limitations of bibliometric analysis methods, this study can only describe the development and evolution of monkeypox in some research fields to a certain extent, which may not fully conform to the actual situation. More accurate conclusions need to be integrated with other analysis methods, including analysis software. Therefore, the results and conclusions drawn in this paper are for reference only.

## Conclusions

This is the first bibliometric study providing detailed information on the published literature on monkeypox between 1975 and 2022 based on Web of Science core database. The increase in the number of monkeypox literature was concentrated after 2003. Over the past 47 years, infection, vaccine and efficacy were the top topic and should be paid more attention in the future study for monkeypox. Contact tracing, testing, surveillance, and vaccination are the main measures since the human monkeypox outbreak in May 2022. The treatment and management targeting human monkeypox deserve further attention.

## Data availability statement

The original contributions presented in the study are included in the article/Supplementary material, further inquiries can be directed to the corresponding authors.

## Author contributions

JYL and LL: study design. JYL, GYL, PLZ, and QZ: data collection. JYL, QZ, and LC: statistical analysis. JYL and GYL: drafting manuscript. LL and LC: revision manuscript. All authors contributed to manuscript revision, read, and approved the submitted version.

## Funding

This study was funded by special funds for the construction of high-level hospitals in Shenzhen Traditional Chinese Medicine Hospital, China Postdoctoral Science Foundation (No. 2022M713649), Guangdong Basic and Applied Basic Research Foundation (2020A1515010758), and the Science and Technology Innovation Projects of Shenzhen (JCYJ 20190812171611467).

## Conflict of interest

The authors declare that the research was conducted in the absence of any commercial or financial relationships that could be construed as a potential conflict of interest.

## Publisher's note

All claims expressed in this article are solely those of the authors and do not necessarily represent those of their affiliated organizations, or those of the publisher, the editors and the reviewers. Any product that may be evaluated in this article, or claim that may be made by its manufacturer, is not guaranteed or endorsed by the publisher.
