# Supplementary material for: Bibliometric analysis of human monkeypox research from 1975 to 2022 and novel prevention and control strategies
Source: Front Public Health. 2022 Sep 27;10:995965. doi: 10.3389/fpubh.2022.995965 (PMC9550883; doi:10.3389/fpubh.2022.995965)
Supplement: Supplementary file 1 [file Data_Sheet_1.docx]

**Table S1. Top 20 cited articles on monkeypox.**

| **Number** | **Title** | **Author** | **Journal** | **Year** | **Total Cited** |
| --- | --- | --- | --- | --- | --- |
| 1 | The detection of monkeypox in humans in the western hemisphere | [Kurt D Reed](https://pubmed.ncbi.nlm.nih.gov/?sort=date&term=Reed+KD&cauthor_id=14736926), [John W Melski](https://pubmed.ncbi.nlm.nih.gov/?sort=date&term=Melski+JW&cauthor_id=14736926), [Mary Beth Graham](https://pubmed.ncbi.nlm.nih.gov/?sort=date&term=Graham+MB&cauthor_id=14736926), et al | New England Journal Of Medicine | 2004 | 347 |
| 2 | The role of evolution in the emergence of infectious diseases | Total CitedRustom Antia , Roland R Regoes, Jacob C Koella, et al | Nature | 2003 | 338 |
| 3 | Pathways to zoonotic spillover | Plowright, K Raina, Parrish, et al | Nature Reviews Microbiology | 2017 | 317 |
| 4 | Live variola virus: considerations for continuing research introduction | [Arvin, A. M](https://xueshu.baidu.com/s?wd=author:(Arvin,%20A.%20M)%20&tn=SE_baiduxueshu_c1gjeupa&ie=utf-8&sc_f_para=sc_hilight=person)，[Patel, D. M](https://xueshu.baidu.com/s?wd=author:(Patel, D. M) &tn=SE_baiduxueshu_c1gjeupa&ie=utf-8&sc_f_para=sc_hilight=person" \t "https://xueshu.baidu.com/usercenter/paper/_blank) | The National Academies Press | 2009 | 289 |
| 5 | Poxvirus tropism | McFadden, G | Nature Reviews Microbiology | 2005 | 285 |
| 6 | Immunogenicity of a highly attenuated mva smallpox vaccine and protection against monkeypox | Patricia L Earl, Jeffrey L Americo, Linda S Wyatt,et al | Nature | 2004 | 261 |
| 7 | A preliminary assessment of silver nanoparticle inhibition of monkeypox virus plaque formation | James V. Rogers, Christopher V. Parkinson, Young W. Choi, et al | Nanoscale Research Letters | 2008 | 258 |
| 8 | Poxvirus genomes: A phylogenetic analysis | C Gubser, S Hué, P Kellam, et al | Journal Of General Virology | 2004 | 254 |
| 9 | Panmicrobial oligonucleotide array for diagnosis of infectious diseases | Palacios G, Quan PL, Jabado OJ, et al | Emerging Infectious Diseases | 2007 | 234 |
| 10 | Diagnosis and management of smallpox | [JG Breman](https://xueshu.baidu.com/s?wd=author:(JG%20Breman)%20&tn=SE_baiduxueshu_c1gjeupa&ie=utf-8&sc_f_para=sc_hilight=person), [DA Henderson](https://xueshu.baidu.com/s?wd=author:(DA%20Henderson)%20&tn=SE_baiduxueshu_c1gjeupa&ie=utf-8&sc_f_para=sc_hilight=person) | New England Journal Of Medicine | 2002 | 233 |
| 11 | The role of wildlife in emerging and re-emerging zoonoses | R G Bengis, FA Leighton, J R Fischer, et al | Revue Scientifique Et Technique-Office International Des Epizooties | 2004 | 214 |
| 12 | An orally bioavailable antipoxvirus compound (st-246) inhibits extracellular virus formation and protects mice from lethal orthopoxvirus challenge | G. Yang, D. C. Pevear, M. H. Davies, et al | Journal Of Virology | 2005 | 213 |
| 13 | An emergent poxvirus from humans and cattle in rio de janeiro state: Cantagalo virus may derive from brazilian smallpox vaccine | [CRA Damaso](https://xueshu.baidu.com/s?wd=author:(CRA%20Damaso)%20&tn=SE_baiduxueshu_c1gjeupa&ie=utf-8&sc_f_para=sc_hilight=person), [JJ Esposito](https://xueshu.baidu.com/s?wd=author:(JJ%20Esposito)%20&tn=SE_baiduxueshu_c1gjeupa&ie=utf-8&sc_f_para=sc_hilight=person), [RC Condit](https://xueshu.baidu.com/s?wd=author:(RC%20Condit)%20&tn=SE_baiduxueshu_c1gjeupa&ie=utf-8&sc_f_para=sc_hilight=person), et al | Virology | 2000 | 207 |
| 14 | Smallpox vaccine-induced antibodies are necessary and sufficient for protection against monkeypox virus | [Yvette Edghill-Smith](https://xueshu.baidu.com/s?wd=author:(Yvette%20Edghill-Smith)%20&tn=SE_baiduxueshu_c1gjeupa&ie=utf-8&sc_f_para=sc_hilight=person), [Hana Golding](https://xueshu.baidu.com/s?wd=author:(Hana%20Golding)%20&tn=SE_baiduxueshu_c1gjeupa&ie=utf-8&sc_f_para=sc_hilight=person), [Jody Manischewitz](https://xueshu.baidu.com/s?wd=author:(Jody%20Manischewitz)%20&tn=SE_baiduxueshu_c1gjeupa&ie=utf-8&sc_f_para=sc_hilight=person), et al | Nature Medicine | 2005 | 205 |
| 15 | Major increase in human monkeypox incidence 30 years after smallpox vaccination campaigns cease in the democratic republic of congo | Anne W Rimoin, Prime M Mulembakani, Sara C Johnston, et al | Proceedings of the National Academy of Sciences of the United States of America | 2010 | 196 |
| 16 | Poxvirus orthologous clusters: Toward defining the minimum essential poxvirus genome | [Chris Upton](https://www.researchgate.net/profile/Chris-Upton), [Stephanie Slack](https://www.researchgate.net/scientific-contributions/Stephanie-Slack-35453437), [Arwen L Hunter](https://www.researchgate.net/scientific-contributions/Arwen-L-Hunter-12335626), et al | Journal Of Virology | 2003 | 194 |
| 17 | Zoonotic poxviruses | Sandra Essbauer, Martin Pfeffer, Hermann Meyer | Veterinary Microbiology | 2010 | 180 |
| 18 | Outbreak of human monkeypox, democratic republic of congo, 1996-1997 | Hutin, Yvan J.F, Williams, et al | Emerging Infectious Diseases | 2001 | 180 |
| 19 | Smallpox dna vaccine protects nonhuman primates against lethal monkeypox | Hooper, W J , Thompson, et al | Journal Of Virology | 2004 | 173 |
| 20 | Human monkeypox: An emerging zoonosis | Daniel B Di Giulio, DrPaul B Eckburg | Lancet Infectious Diseases | 2004 | 172 |

**Table S2** The top 20 most productive institutions in monkeypox research

| **Number** | **Institutions** | **Full name of the institutions** | **Article counts** | **Total number of citations** | **Average number of citations** |
| --- | --- | --- | --- | --- | --- |
| 1 | ctr dis control & prevent | Centers for Disease Control Prevention USA | 165 | 4838 | 29.32 |
| 2 | niaid | NIH National Institute of Allergy & Infectious Diseases | 71 | 2165 | 30.49 |
| 3 | st louis univ | Saint Louis University | 33 | 1245 | 37.72 |
| 4 | univ kinshasa | Universite de Kinshasa | 29 | 250 | 8.62 |
| 5 | who | World Health Organization | 27 | 1363 | 50.48 |
| 6 | robert koch inst | Robert Koch Institute | 25 | 658 | 26.32 |
| 7 | state res ctr virol & biotechnol vector | State Research Center of Virology & Biotechnology VECTOR | 23 | 583 | 25.35 |
| 8 | univ calif los angeles | University of California Los Angeles | 23 | 904 | 39.30 |
| 9 | us fda | US Food & Drug Administration (FDA) | 23 | 650 | 28.26 |
| 10 | nih | National Institutes of Health (NIH) - USA | 22 | 1312 | 59.64 |
| 11 | inst natl rech biomed | Institute Nacional de Recherche Biomedical | 21 | 173 | 8.24 |
| 12 | katholieke univ leuven | Katholieke Univ Leuven | 21 | 1107 | 52.71 |
| 13 | oregon hlth & sci univ | Oregon Health & Science University | 19 | 749 | 39.42 |
| 14 | univ penn | University of Pennsylvania | 19 | 807 | 42.47 |
| 15 | univ fed minas gerais | Universidade Federal de Minas Gerais | 17 | 208 | 12.24 |
| 16 | univ florida | University of Florida | 17 | 698 | 41.06 |
| 17 | stanford univ | Stanford University | 16 | 884 | 55.25 |
| 18 | univ wisconsin | University of Wisconsin Madison | 16 | 237 | 14.81 |
| 19 | emory univ | Emory University | 15 | 669 | 44.60 |
| 20 | inst pasteur | Institut Pasteur Paris | 15 | 280 | 15.40 |
